# Chemical journey of somatic cells to pluripotency

**DOI:** 10.1186/s13619-022-00126-7

**Published:** 2022-08-03

**Authors:** Deepti Abbey

**Affiliations:** Institute for Stem Cell Science and Regenerative Medicine, GKVK – Post, Bellary Road, Bangalore, 560065 India

## Abstract

Reprogramming somatic cells to pluripotent stem cells has revolutionized the biomedical field by providing enormous hopes and opportunities for the regeneration of tissues and organs for transplantation. Using a small molecule cocktail of epigenetic modifiers and cell signalling inhibitors, a chemical-based easy and controllable technique for converting human somatic cells into chemically induced pluripotent stem cells was recently reported (Guan, Nature 605:325–31, 2022). This novel approach offers well-defined, safe, simple, easy, and clinical-grade manufacturing strategies for modifying the fate of human cells required for regenerative therapeutics.

## Main text

Since Yamanaka’s team identified four key factors in 2006–2007 (Takahashi and Yamanaka 2006), researchers have been looking for a chemical cocktail to reduce the viral load involved in somatic cell reprogramming ever since. Episomal, RNP, protein-based, and miRNA techniques are among the others that have been documented, but each has its drawbacks and can only reprogram cells with an efficiency of 0.01 to 1% (al Abbar et al. 2020). Chemical inducers that target cell signalling pathways and epigenetic modifiers have been shown to reprogram mouse somatic cells and transdifferentiate (also known as lineage reprogram) human and mouse cells. Despite this, previous attempts to reprogram human somatic cells have failed, because these cells maintain a stable epigenome (Takeda et al. 2018; Yang et al. 2022; Xu, Du, and Deng 2015).

Following leads from mouse somatic cell chemical reprogramming and chemical library screens, (Guan et al., 2022) the authors reported a cocktail of 16 small molecules consisting of CHIR99021, 616,452, TTNPB, Y27632, ABT869, SAG, JNKIN8, 5-azacytidine, tranylcypromine, valproic acid, DZNep, EPZ004777, UNC0379, and PD0325901 required in stage-specific manner for conversion of fibroblasts to epithelial-like cells and later for activating pluripotency master regulator, Oct 4. The colonies obtained on chemical reprogramming (designated as human chemically iPS cells (hCiPS)) displayed typical hES cell morphology, doubling time, transcriptomic and epigenomic profiles.

The authors used a similar chemical reprogramming approach on adult somatic cells such as adipose-derived mesenchymal stromal cells and adult skin dermal fibroblasts to generate hCiPS with efficiencies ranging from 0.21 ± 0.07% to 2.56 ± 0.63%. Adult somatic-cell-derived hCiPS cells were able to produce cell types from three germ layers in vivo and in vitro, and their transcriptome and epigenetic profiles were similar to those of hES cells.

During the reprogramming trajectory in the current study using human cells, various cell states known as an intermediate plastic state, an extraembryonic endoderm-like (XEN-like) state, and naive pluripotency state were identified, as previously reported from the same group in mouse cells (Zhao et al. 2015; Li et al. 2017). In contrast, primitive streak-like intermediates are observed in OSKM-induced reprogramming, whereas the XEN-like state is exclusive to chemical reprogramming and is rarely documented (Parenti et al. 2016). Interestingly, authors reported that during the intermediate plastic state, cells were reprogrammed to acquire characteristics of developing human limb bud cells that are similar to amphibian (axolotl) limb regeneration, where regeneration-like gene regulatory programs governing embryonic limb development are reactivated which is indispensable for acquiring cell pluripotency.

In contrast to studies of direct chemical transdifferentiation, in which cells do not transition to any transitory pluripotent state, the current investigation highlighted the establishment of an intermediate plastic population that resembles genes relevant to limb development across species. This plastic state is reported to have open chromatin and reduced DNA methylation at a global level.

One of the limitations of CiPS cells is the longer duration of reprogramming of 5–6 weeks vs 2–4 weeks for viral and episomal approaches. However, more comprehensive analysis, and additional optimizations are required to test the proposed cocktail with other somatic cells from humans and different species for reprogramming efficiency. Along similar lines, a recent report describes the use of three small molecules alone to generate ‘totipotent’ mouse cells from pluripotent stem cells (Hu et al. 2022). Furthermore, more insight is required to determine whether the small molecules used have potential off target effects prior to evaluating their clinical implications. Future research on retention and the role of epigenetic memory in chemical reprogramming would open further avenues for the regenerative field by providing an opportunity to select appropriate starting cell material for therapies.

## Conclusions

**Fig. 1:**
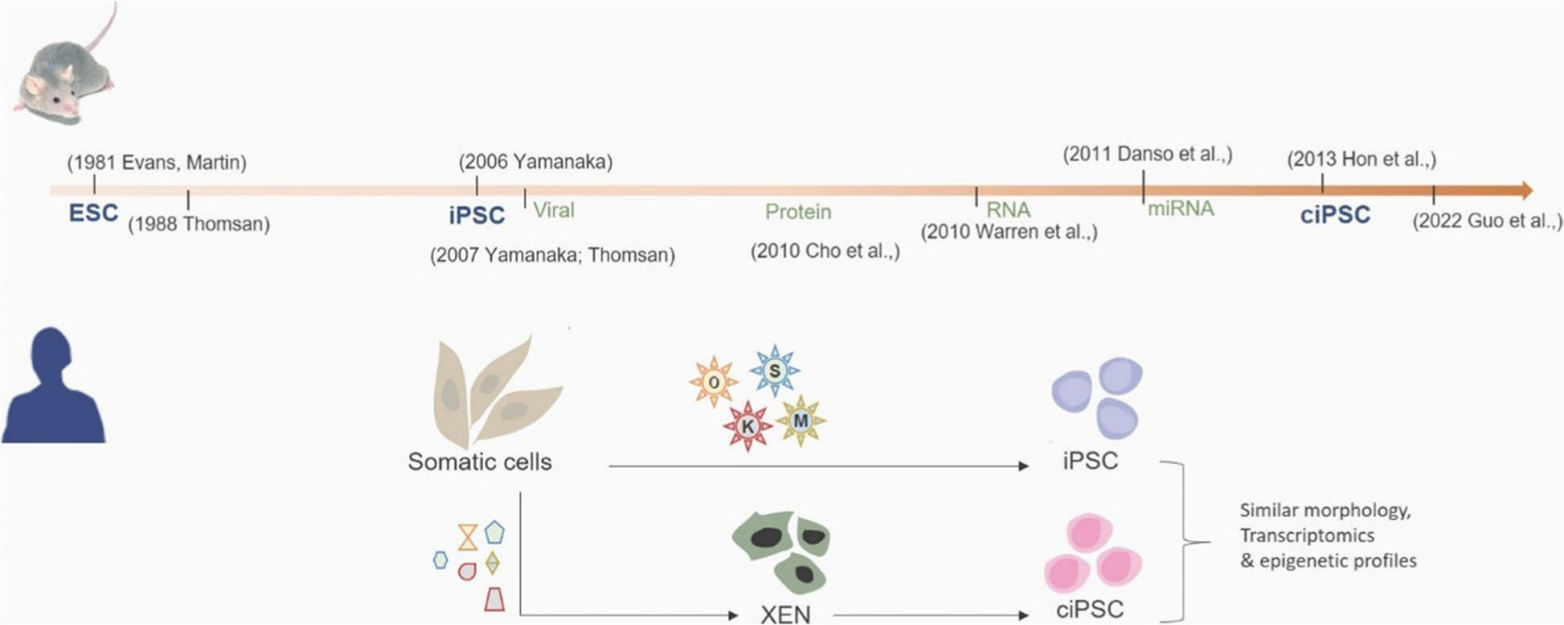
Evolution of pluripotent stem cells using different reprogramming approaches

It is intriguing to note that the authors of the current study employed a chemical cocktail that inhibits global DNMT methylation, histone acetylation, histone methylation, and other major cell signalling pathways, which in turn provides an open (environment) chromatin accessible to a variety of transcription factors and erases the cell’s history in order to write a new paradigm. The proposed approach has significant promise for stimulating in vivo repair and regeneration of patients’ endogenous cells. Furthermore, the method minimizes the risk of tumorigenesis associated with viral methods (Fig. 1).

## Data Availability

Not applicable.
